# Benefit and safety of capecitabine after resection of biliary tract cancer: a real-world study with propensity score matching

**DOI:** 10.3389/fonc.2025.1697673

**Published:** 2025-12-16

**Authors:** Hugo Roussel, Sophie Le Joncour, Valerie Aurillac, Arthur Marichez, Jean Frederic Blanc, Laurence Chiche, Christophe Laurent, Marie Decraecker

**Affiliations:** 1 Pharmacy Unit, Hôpital Haut Lévêque, Bordeaux University Hospital, Pessac, France; 2 Oncology Unit, Hôpital Haut Lévêque, Bordeaux University Hospital, Pessac, France; 3 Digestive Surgery Unit, Hôpital Haut Lévêque, Bordeaux University Hospital, Pessac, France

**Keywords:** adjuvant, biliary tract, capecitabine, disease-free survival, propensity score

## Abstract

Biliary tract cancer (BTC) is a high mortality disease, with few patients eligible for resection. Even post-surgery, recurrence rates remain high. The benefit of capecitabine as adjuvant therapy is uncertain, as seen in the BILCAP trial results. This study aimed to evaluate the impact of capecitabine in a real-world setting among patients who underwent surgery for localized BTC. This retrospective observational study compared a cohort of patients who received adjuvant capecitabine with an observation-only cohort. Recurrence-free survival (RFS) and overall survival (OS) were estimated and compared between the two groups, with propensity score matching to minimize selection bias. Differences in RFS were also analyzed between patients completing the full capecitabine protocol and those on an adapted protocol. From January 2017 to May 2023, 117 patients were included in the observation cohort, and 43 patients were included in the capecitabine cohort. After propensity score matching, no difference in RFS was found (HR = 0.700, *P* = 0.34). Additionally, RFS was similar between patients who completed the full capecitabine protocol and those who received an adapted protocol (HR = 0.265, *P* = 0.19). This study showed no clear benefit of capecitabine; further research is needed to improve survival outcomes.

## Introduction

Biliary tract cancer (BTC) is a heterogeneous group of diseases with increasing incidence in Western countries. It is classified based on anatomical location and includes intrahepatic cholangiocarcinoma (iCCA), perihilar cholangiocarcinoma (pCCA), extrahepatic cholangiocarcinoma (eCCA), and gallbladder cancer (GBC). BTC has a poor prognosis, with high mortality rates and low overall survival (OS); the 5-year OS for BTC is reportedly only 17%, regardless of disease stage (1). Fewer than 12% of patients are candidates for tumor resection, one of the few potentially curative treatments (2). Most patients are diagnosed at an advanced stage of disease, when palliative care is the only option. Even for those undergoing surgical resection with curative intent, survival outcomes are concerning. For example, the median recurrence-free survival (RFS) after resection of iCCA is less than 2 years (3).

Several clinical trials have explored the efficacy of adjuvant therapies, but none have shown a clear benefit (4). The BILCAP trial compared capecitabine (oral fluorouracil, Xeloda^®^; Roche, Basel, Switzerland) with observation after resection of BTC. The trial showed slight superiority of capecitabine in terms of the secondary endpoint, RFS, but only in the per-protocol analysis. This benefit was statistically significant only after adjusting for prognostic factors such as nodal status, disease grade, and sex. There was no clear benefit of capecitabine in terms of the primary endpoint, OS, regardless of tumor location (5). However, recently updated long-term results of the trial demonstrated a significant improvement in OS in the capecitabine cohort (CC), with a 16-month increase in survival (CC: 52.3 months, observation cohort [OC]: 36.1 months; hazard ratio [HR] = 0.79, 95% confidence interval [CI] = 0.63–1.00) (6).

As a result of these findings, the French guidelines were updated in November 2022 to recommend capecitabine as standard adjuvant chemotherapy after curative resection for localized BTC (7). The standard recommended dosage is 1250 mg/m² twice daily for eight cycles (6 months of treatment). Patients typically receive capecitabine for 2 weeks, followed by a 7-day break, forming a 3-week treatment cycle.

However, capecitabine is associated with serious side effects, including hand–foot syndrome, neutropenia, stomatitis, diarrhea, nausea, and vomiting. In the BILCAP study, 44% of patients in the capecitabine group experienced at least one grade 3 toxicity (5). Among these toxicities, hand–foot syndrome was the most common, affecting 20% of patients (5).

Clinical pharmacy is a health science discipline focused on optimizing medication therapy and promoting health and disease prevention (8). Clinical pharmacists play a key role in managing the adverse effects of oral chemotherapy, ensuring medication safety, and improving patient compliance. Their involvement in hospital departments has demonstrated both clinical and economic benefits (9). The 2021 German AMBORA trial showed that intensified clinical pharmacological and pharmaceutical care was able to improve medication safety (10). This trial utilized multidisciplinary pathways involving pharmacists, nurses, and physicians, which enhanced patients’ understanding of their treatment and drug interactions. Nurses monitored patients’ drug tolerance at home, whereas clinical pharmacists provided detailed guidance on medication use and communicated with community pharmacies to ensure treatment continuity.

The results of the BILCAP study, along with the iatrogenic profile of capecitabine, prompted further analysis of its benefits in the adjuvant setting and real-world practice. However, no such study has been conducted to date. The present study aimed to assess the real-world benefit of adjuvant capecitabine in patients who underwent resection of localized BTC and were followed at our university hospital.

## Patients & methods

### Patients

This single-center, retrospective, observational study included all consecutive patients aged > 18 years who underwent curative resection of cholangiocarcinoma (CCA) or GBC at the Digestive Surgery Unit of Bordeaux University Hospital from 1 January 2017 to 31 May 2023. Patients with a histological diagnosis of ampulloma, pancreatic cancer, or hepatocellular carcinoma, as well as those with concomitant active cancer, were excluded. The study population was divided into two groups: one group consisted of patients who received adjuvant capecitabine treatment, and the other group comprised patients who were monitored postoperatively without specific treatment.

### Capecitabine treatment

In accordance with the BILCAP protocol, patients initiated capecitabine treatment within 16 weeks after BTC resection. To meet the minimum follow-up requirement of 1 year, patients were required to begin treatment before 31 May 2023. The treatment was administered in 3-week cycles (2 weeks of capecitabine followed by a 1-week break). The dosage was 1250 mg/m² twice daily; reductions were implemented for patients with poor tolerance or elevated uracil levels detected in blood tests. Patients who underwent dose modifications or early treatment termination were still included in the statistical analyses. Before treatment initiation, patients met with both a nurse and a pharmacist, who provided detailed explanations of the treatment regimen, potential side effects, and handling precautions based on the iatrogenic profile of capecitabine.

### Statistical analysis

Descriptive results are presented as medians with interquartile ranges (IQRs) for quantitative data and as percentages for qualitative data. Quantitative variables were compared using the Wilcoxon–Mann–Whitney test. Qualitative variables were compared using Fisher’s exact test for subgroups of five or less patients; for subgroups of more than five patients, the chi-square test was used. The threshold for statistical significance was set to *P* < 0.05.

OS and RFS were compared using HRs with 95% CIs, estimated through a Cox proportional hazards model. Univariate Cox regression analyses were performed to assess the relationship between individual variables and survival, and multivariate Cox analysis was conducted to evaluate associations between groups of variables and survival. The proportional hazards assumption was verified for all variables in both univariable and multivariable Cox models using a correlation test between Schoenfeld residuals and time. Significant variables identified through univariate Cox regression were included in the multivariate analysis. Kaplan–Meier survival estimates were then used to analyze survival in both cohorts. A log-rank test was subsequently conducted to compare the Kaplan–Meier survival curves.

Propensity scores (PSs) were calculated using logistic regression. R0 resection and nodal status are established determinants of treatment decisions prior to the French guideline revision. The propensity score model was adjusted for these two variables, along with all other covariates that showed a statistically significant difference between the groups. Matching was performed between the CC and OC using the PSMATCH procedure in SAS software (SAS Institute, Cary, NC, USA). Matching was performed using the nearest-neighbor method without replacement, with a caliper of 0.05 on the propensity score. Survival analyses were then conducted on the matched cohort. The quality of the PS matching was assessed using standardized mean differences (SMD) for continuous variables and differences in proportions for categorical variables, before and after matching. All analyses were performed using SAS v9.4 (SAS Institute).

### Primary and secondary endpoints

The primary endpoint was RFS, defined as the time from surgery to the date of the first multidisciplinary team discussion confirming disease recurrence. Tumor recurrence was monitored using computed tomography or magnetic resonance imaging scans. RFS was compared between the CC and OC.

The secondary endpoint was OS, defined as the time from surgery to death from any cause. Follow-up time was defined as the interval between surgery and the last consultation or communication with the patient. In patients without disease recurrence, the date of the last follow-up was used for survival evaluation.

Patient tolerance of treatment was assessed using the Common Terminology Criteria for Adverse Events. Survival analyses were conducted between patients treated with an adapted protocol (AP) (dose reduction, treatment break, or early termination) and those who completed the full protocol (complete protocol [CP]). An additional analysis was performed to assess the impact of early termination on survival by comparing patients treated with an AP (dose reduction, treatment break due to toxicity), with or without early termination.

## Results

### Patients

**Figure 1:**
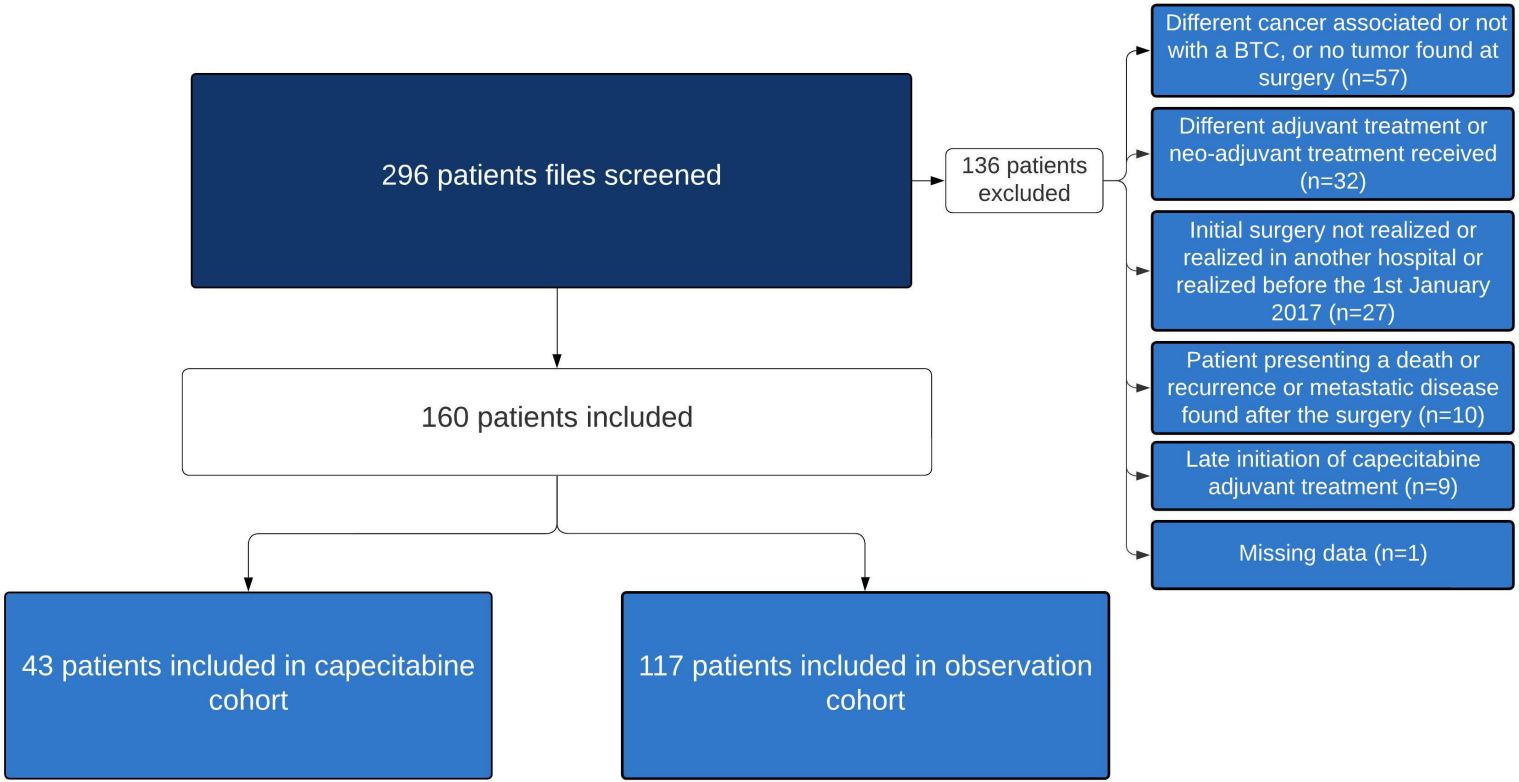
Study profile.

In total, 296 patients underwent tumor resection surgery for suspected BTC from 1 January 2017 to 31 May 2023 (**Figure 1**). Of these patients, 136 were excluded for the following reasons: 57 (41.9%) had other cancers, tumor absence discovered during surgery, or tumors not associated with BTC; 32 (23.5%) received adjuvant treatments other than capecitabine or neoadjuvant treatment; 27 (19.9%) did not undergo surgery, underwent surgery at another hospital, or underwent surgery before 1 January 2017; 10 (7.4%) died or developed recurrence after surgery; 9 (6.6%) underwent late initiation of capecitabine treatment not allowing for the minimum one-year follow-up period; and 1 (0.7%) had missing data. Ultimately, 117 patients were included in the OC; 43 patients were included in the CC.

In the global population (GP, made up of all treated and untreated patients), 60 patients were women (GP: 37.5%, OC: 39.3%, CC: 32.6%; *P* = 0.43) (**Table 1**). There were no significant differences in the median age at diagnosis (GP: 69 years, OC: 70 years, CC: 68 years; *P* = 0.08) or median body mass index (GP: 24 kg/m², OC: 24.1 kg/m², CC: 25.2 kg/m²; *P* = 0.25). The distribution of cancer types (iCCA, eCCA, pCCA, and GBC) was similar between the cohorts (*P* = 0.76). Likewise, ECOG scores were comparable between the cohorts (*P* = 0.36). However, there were significantly more patients with positive nodes in the CC than in the OC (CC: 57.1%, OC: 31.6%; *P* = 0.005), as well as a higher rate of R1 resection (CC: 39.5%, OC: 23.1%; *P* = 0.04). The median follow-up was similar between the cohorts (OC: 21 months, CC: 19 months; *P* = 0.67). No significant differences were detected in mortality, disease recurrence, percentage of living patients at the last follow-up, percentage of patients in remission, or mortality after recurrence between the cohorts (*P* = 0.13).

**Table 1:** Patient characteristics.

Capecitabine vs. observation	Capecitabine cohort	n (%) or median (interquartile range)	Observation cohort	n (%) or median (interquartile range)	*P*
Female sex	43	14 (32.6)	117	46 (39.3)	0.43
BMI, kg/m^2^	43	25.2 (21.3–27.9)	117	24.1 (21.7–26.9)	0.25
Age at diagnosis, years	43	68 (56–73)	117	70 (63–74)	0.08
Follow-up, months	43	19 (10–33)	117	21 (11–36)	0.67
ECOG score at diagnosis	43		114		0.36
*0*		32 (74.4)		92 (80.7)	
*1*		9 (20.9)		20 (17.5)	
*≥2*		2 (4.7)		2 (1.8)	
Underlying liver disease	43	6 (13.9)	117	18 (15.4)	0.82
Localization	43		117		0.76
*iCCA*		16 (37.2)		49 (41.9)	
*pCCA*		11 (25.6)		26 (22.2)	
*eCCA*		14 (32.6)		32 (27.4)	
*GBC*		2 (4.7)		10 (8.5)	
Tumor size, cm	43	3.8 (2.4–6.5)	107	3.0 (2.0–6.0)	0.33
Positive node status	42	24 (57.1)	95	30 (31.6)	**0.005**
Degree of differentiation	42		103		0.30
*Poorly differentiated*		3 (7.1)		19 (18.4)	
*Moderately differentiated*		23 (54.8)		46 (44.7)	
*Well to very well differentiated*		16 (38.1)		38 (36.9)	
Presence of invasion	43	35 (81.4)	117	91 (77.8)	0.62
R0 resection	43	26 (60.5)	117	90 (76.9)	**0.04**
Patient health at last follow-up	43		117		0.13
*Death*		11 (25.6)		40 (34.2)	
*Lost to follow-up*		3 (7.0)		14 (12.0)	
*Alive*		6 (14.0)		27 (23.1)	
*Remission*		7 (16.3)		10 (8.5)	
*Recurrence*		16 (37.2)		26 (22.2)	

Data are presented as n (%) or median (interquartile range), bold p-values indicate statistically significant differences (p < 0.05). BMI, body mass index; ECOG, Eastern Cooperative Oncology Group; iCCA, intrahepatic cholangiocarcinoma; pCCA, perihilar cholangiocarcinoma; eCCA, extrahepatic cholangiocarcinoma; GBC, gallbladder cancer.

### Capecitabine treatment

The median time to initiation of capecitabine treatment was 2.1 months (IQR: 1.8–2.5) (**Table 2**). Of the 43 patients in the CC, 40 had documented treatment initiation; fewer than 10% began at a reduced dosage due to poor general condition (n = 2) or renal failure (n = 1). Uracil blood tests were performed in 22 patients (51.2%). Fewer than half of the patients (n = 16, 40%) experienced treatment interruptions, primarily due to side effects such as hand–foot syndrome (n = 7, 43.75%), poor general condition (n = 5, 31.25%), digestive toxicity (n = 1, 6.25%), infection (n = 1, 6.25%), neuropsychiatric syndrome (n = 1, 6.25%), or a combination of hand–foot syndrome and digestive toxicity (n = 1, 6.25%).

**Table 2:** Capecitabine treatment characteristics.

Capecitabine cohort	Global population (n = 43)	n (%) or median (interquartile range)
Time to postoperative initiation of capecitabine, months	39	2.1 (1.8–2.5)
Initial dosage decreased	33	3 (9.1)
Normal uracil blood test result	22	22 (100)
Dosage decreased	31	4 (12.9)
Treatment paused	31	16 (51.6)
Digestive toxicity	16	1 (6.25)
Hand–foot syndrome	16	8 (50.0)
Early termination of treatment	39	17 (43.6)
Reason for early termination	39	17 (43.6)
*Death unrelated to disease*	17	1
*Cancer recurrence*	17	7
*Side effects*	17	5 (55.6)
*No known cause*	17	4 (44.4)
Inclusion in tripartite care pathway	42	9 (21.4)

Data are presented as n (%) or median (interquartile range).

Of the 39 patients with documented treatment cycles, 9 (23.1%) discontinued treatment early, mainly due to adverse effects (55.6%; hand–foot syndrome in 1, anemia in 1, phlycten in 1, and poor general condition in 2) or unknown reasons (n = 4, 44,4%). Fewer than one-quarter of patients (20.9%) consulted a pharmacist during treatment.

### Survival

**Figure 2:**
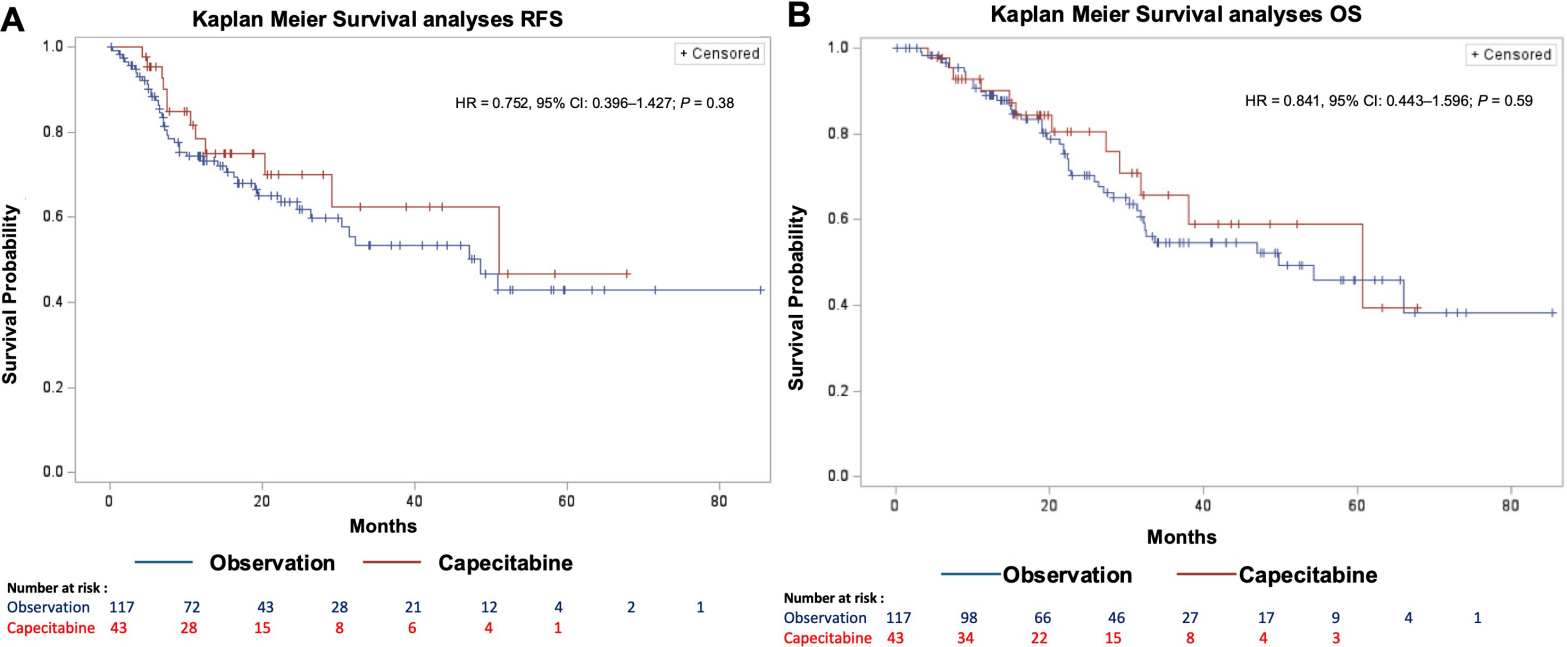
Multivariate recurrence free and overall survival. [**(A)** RFS; **(B)** OS; HR, Hazard ratio; CI, Confidence interval; RFS, Recurrence free survival; OS, Overall survival].

At the time of assessment, the median RFS for the GP was 14.0 months (IQR: 6.9–27.3); there was no significant difference between the two cohorts (CC: 13.8 months, OC: 14.1 months; HR = 0.752, 95% CI = 0.396–1.427; *P* = 0.38) (**Figure 2**). The median OS was 22.3 months (IQR: 12.6–37.1), again showing no significant difference between the cohorts (CC: 20.3 months, OC: 22.5 months; HR = 0.841, 95% CI = 0.443–1.596; *P* = 0.59).

When stratified by tumor localization, patients with intrahepatic cholangiocarcinoma (iCCA) derived no apparent benefit from adjuvant capecitabine in relapse-free survival (RFS: iCCA CC, 11.3 months; OC, 10.0 months; HR = 0.311; 95% CI, 0.072–1.339; P = 0.09) or overall survival (OS: iCCA CC, 20.2 months; OC, 19.1 months; HR = 0.335; 95% CI, 0.078–1.444; P = 0.12).

Similarly, no significant differences were observed between cohorts among patients with extrahepatic cholangiocarcinoma (eCCA) for either RFS (eCCA CC, 15.3 months; OC, 20.9 months; HR = 0.995; 95% CI, 0.385–2.572; P = 0.99) or OS (eCCA CC, 23.3 months; OC, 30.0 months; HR = 1.089; 95% CI, 0.420–2.825; P = 0.86).

Among patients with perihilar cholangiocarcinoma (pCCA), RFS (pCCA CC, 13.8 months; OC, 14.7 months; HR = 1.153; 95% CI, 0.286–4.646; P = 0.84) and OS (pCCA CC, 22.8 months; OC, 23.8 months; HR = 1.165; 95% CI, 0.290–4.675; P = 0.83) were also comparable between groups.

Finally, for gallbladder cancer (GBC), no significant differences were detected between cohorts in RFS (GBC CC, 12.8 months; OC, 11.0 months; HR = 1.457; 95% CI, 0.140–15.112; P = 0.75) or OS (GBC CC, 12.8 months; OC, 17.0 months; HR = 2.210; 95% CI, 0.196–24.967; P = 0.51).

### Survival analysis after PS matching

The propensity score was adjusted for R0 resection and nodal status. These variables were the only ones that significantly differed between groups before matching and are major determinants in the decision to administer adjuvant treatment, particularly prior to the 2022 French guideline update and BILCAP trial sensitivity analyses. Other potential confounders such as sex were not included in the matching process because of the risk of overfitting given the sample size. The matching procedure reduced the standardized mean differences (SMD) for the variables “resection” and “nodal status” from 1.49 and 1.96 before matching to 0 for both variables after matching (**Table 3**). After matching at a 1:1 ratio, the population was reduced to 84 patients (42 per cohort).

**Table 3:** Propensity score comparison.

Propensity score comparison	Capecitabine cohort	Propensity score (IQR)	Observation cohort	Standardized mean differences
Before matching	42	0.38 (0.30-0.38)	95	0.66
* Female sex*	43		117	0.48
* BMI, kg/m^2^*	43		117	0.14
* Age at diagnosis, years*	43		117	0.36
* Follow-up, months*	43		117	0.12
* ECOG score at diagnosis*	43		117	0.70
* Underlying liver disease*	43		117	0.10
* Localization*	43		117	0.34
* Tumor size, cm*	43		107	0.10
* Positive node status*	42		95	1.96
* Degree of differentiation*	42		103	0.15
* Presence of invasion*	43		117	0.45
* R0 Resection*	43		117	1.49
* Patient health at last follow-up*	43		117	0.57
After matching	42	0.50 (0.50–0.50)	42	0
* Female sex*	42		42	0.98
* BMI, kg/m^2^*	42		42	0.57
* Age at diagnosis, years*	42		42	0.05
* Follow-up, months*	42		42	0.26
* ECOG score at diagnosis*	42		41	0.06
* Underlying liver disease*	42		42	0.36
* Localization*	42		42	0.53
* Tumor size, cm*	42		37	0.27
* Positive node status*	42		42	0
* Degree of differentiation*	41		34	0.26
* Presence of invasion*	42		42	0
* R0 Resection*	42		42	0
* Patient health at last follow-up*	42		42	0.84

Data are presented as propensity score value (interquartile range). IQR, interquartile range.

**Figure 3:**
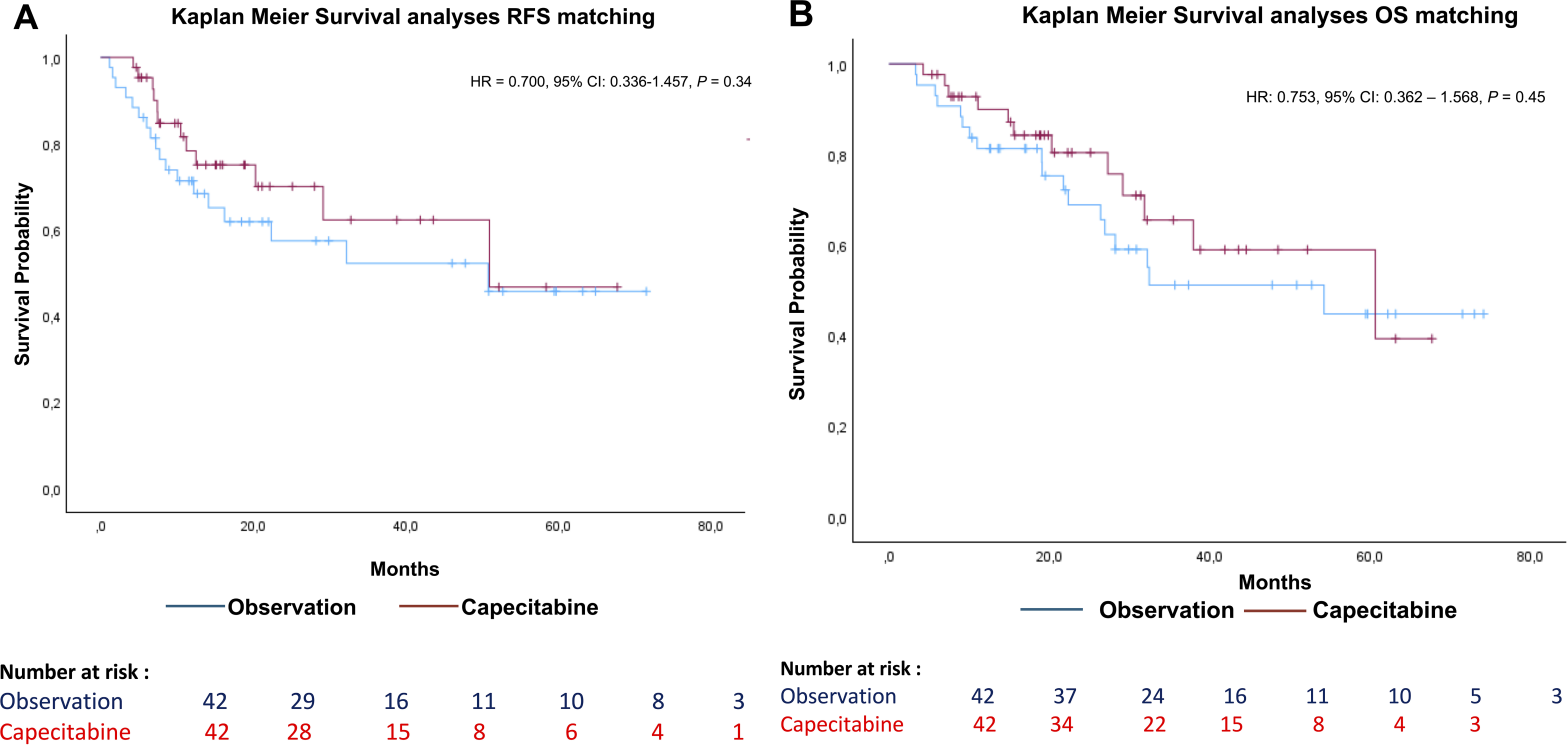
Recurrence free and overall survival after matching population on nodal status and resection. [**(A)** RFS; **(B)** OS; HR, Hazard ratio; CI, Confidence interval; RFS, Recurrence free survival; OS, Overall survival].

After matching, no significant difference in RFS was observed between the two cohorts (CC: 13.8 months, OC: 13.6 months; HR = 0.700, 95% CI = 0.336–1.457; *P* = 0.34) (**Figure 3**). Similarly, OS did not significantly differ after matching (CC: 20.3 months, OC: 22.3 months; HR = 0.753, 95% CI = 0.362–1.568; *P* = 0.45).

### Prognosis

Univariate Cox regression showed that three variables were significantly associated with RFS: median tumor size (HR = 1.127, 95% CI = 1.038–1.223; *P* = 0.0042), number of positive nodes (HR = 2.302, 95% CI = 1.297–4.085; *P* = 0.0042), and tumor differentiation (HR = 0.494, 95% CI = 0.265–0.923; *P* = 0.03). Multivariate analysis identified tumor size (HR = 1.118, 95% CI = 1.026–1.218; *P* = 0.01) and node positivity (HR = 2.536, 95% CI = 1.385–4.643; *P* = 0.0026) as factors significantly associated with survival (**Table 4**).

**Table 4:** RFS univariate and multivariate cox regression.

Covariate	N	WALD *P-value*	HR (95% CI)
Female sex	160	0.44	1.234 (0.722–2.109)
BMI	160	0.82	1.008 (0.944–1.076)
Median age at diagnosis	160	0.07	1.028 (0.998–1.060)
ECOG score at diagnosis (1,2,3 vs 0)	157	0.34	0.693 (0.327–1.470)
Underlying liver disease	160	0.82	1.087 (0.530–2.231)
Localization	160	0.92	1.013 (0.773–1.327)
Median tumor size	150	**0.0042**	1.127 (1.038–1.223)
Positive node status	137	**0.0044**	2.302 (1.297–4.085)
Degree of differentiation (well and very well vs moderately and poorly differentiated)	145	**0.03**	0.494 (0.265–0.923)
Presence of invasion	160	0.47	1.266 (0.665–2.409)
R0 resection	160	0.07	1.667 (0.953–2.915
Multivariate Cox Regression	127	**<0.0004**	
Median tumor size		**0.01**	1.118 (1.026–1.218)
Positive node status		**0.0026**	2.536 (1.385–4.643)

Bold p-values indicate statistically significant differences (p < 0.05). BMI, body mass index; ECOG, Eastern Cooperative Oncology Group; HR, Hazard ratio; CI, Confidence interval.

Univariate Cox regression analysis of OS identified four significant risk factors: median age at diagnosis (HR = 1.037, 95% CI = 1.004–1.071; *P* = 0.03), tumor size (HR = 1.136, 95% CI = 1.038–1.243; *P* = 0.0056), node positivity (HR = 2.000, 95% CI = 1.130–3.541; *P* = 0.02), and degree of tumor differentiation (HR = 1.784, 95% CI = 1.019–3.125; *P* = 0.04). Multivariate analysis revealed median age (HR = 1.046, 95% CI = 1.012–1.082; *P* = 0.0083), node positivity (HR = 2.029, 95% CI = 1.144–3.598; *P* = 0.01), and surgical resection grade (HR = 2.171, 95% CI = 1.203–3.919; *P* = 0.01) as factors significantly associated with mortality (**Table 5**).

**Table 5:** OS univariate and multivariate cox regression.

Covariate	N	Wald *P-value*	HR (95% CI)
Female sex	160	0.40	1.260 (0.737–2.154)
BMI	160	0.98	1,001 (0.935–1.070)
Median age at diagnosis	160	**0.03**	1.037 (1.004–1.071)
ECOG score at diagnosis (1,2,3 vs 0)	157	0.50	0.770 (0.363–1.637)
Underlying liver disease	160	0.72	1.141 (0.555–2.345)
Localization	160	0.60	1.077 (0.815–1.423)
Median tumor size	150	**0.0056**	1.136 (1.038–1.243)
Positive node status	137	**0.02**	2,000 (1.130–3.541)
Degree of differentiation (well and very well vs moderately and poorly differentiated)	145	**0.02**	0.701 (0.513–0.957)
Presence of invasion	160	0.39	1.324 (0.697–2.515)
R0 resection	160	**0.04**	1.784 (1.019–3.125)
Multivariate Cox Regression	137	**<0.0003**	
Median age at diagnosis		**0.0083**	1.046 ( 1.012-1.082)
Positive node status		**0.01**	2.029 (1.144-3,598)
R0 resection		**0.01**	2.171 (1.203-3.919)

Data are presented as n (%) or median (interquartile range). Bold p-values indicate statistically significant differences (p < 0.05). BMI, body mass index; ECOG, Eastern Cooperative Oncology Group; HR, Hazard ratio; CI, Confidence interval.

The proportional hazards assumption was verified for all variables using a correlation test between Schoenfeld residuals and time, and the absence of significant correlation (p > 0.05) confirmed that the assumption was satisfied for all variables in both univariate and multivariate analysis.

### Impact of adapting treatment

**Figure 4:**
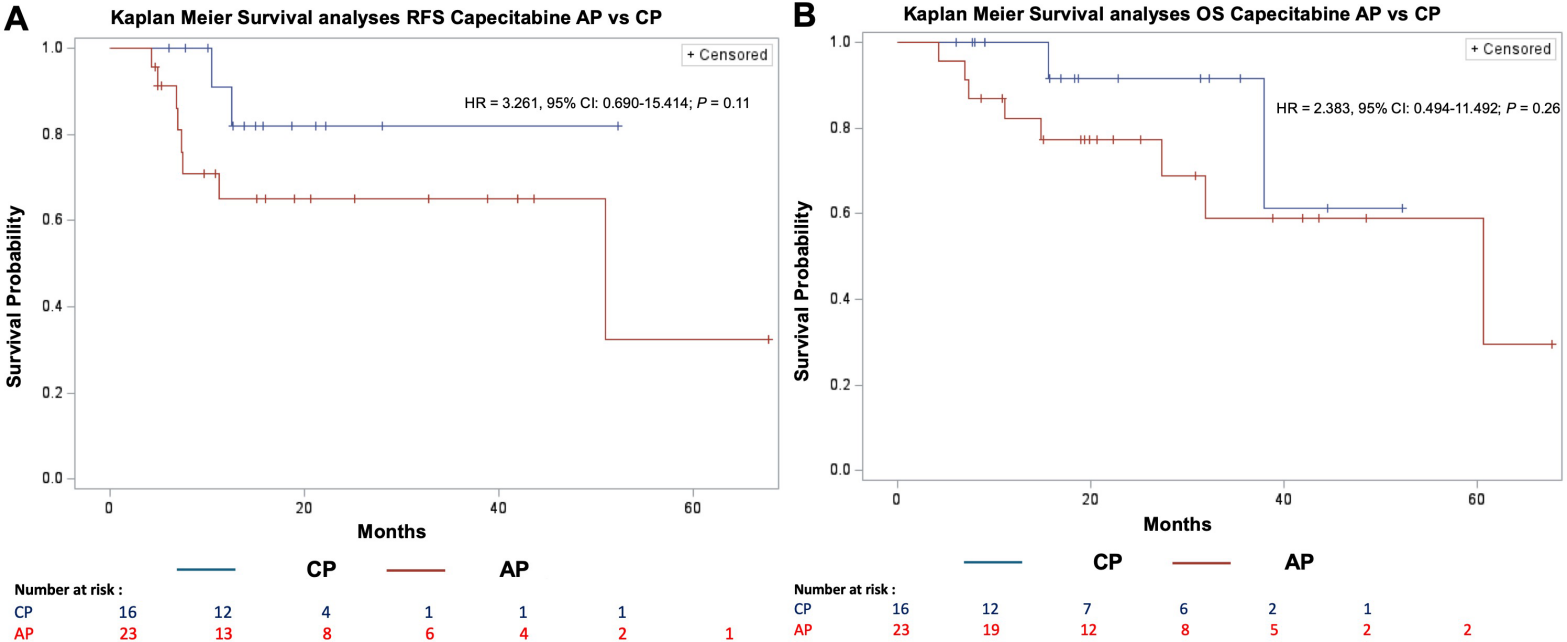
Recurrence free and overall survival after AP or CP treatment. [**(A)** RFS; **(B)** OS; HR, Hazard ratio; CI, Confidence interval; RFS, Recurrence free survival; OS, Overall survival; AP, Adapted protocol; CP, Complete protocol].

No significant difference in RFS was observed between the AP and CP cohorts (AP: 11.2 months, CP: 13.2 months; HR = 3.261, 95% CI = 0.690–15.414; *P* = 0.11) (**Figure 4**). Similarly, there was no significant difference in median OS (AP: 20.6 months, CP: 18.5 months; HR = 2.383, 95% CI = 0.494–11.492; *P* = 0.26).

**Figure 5:**
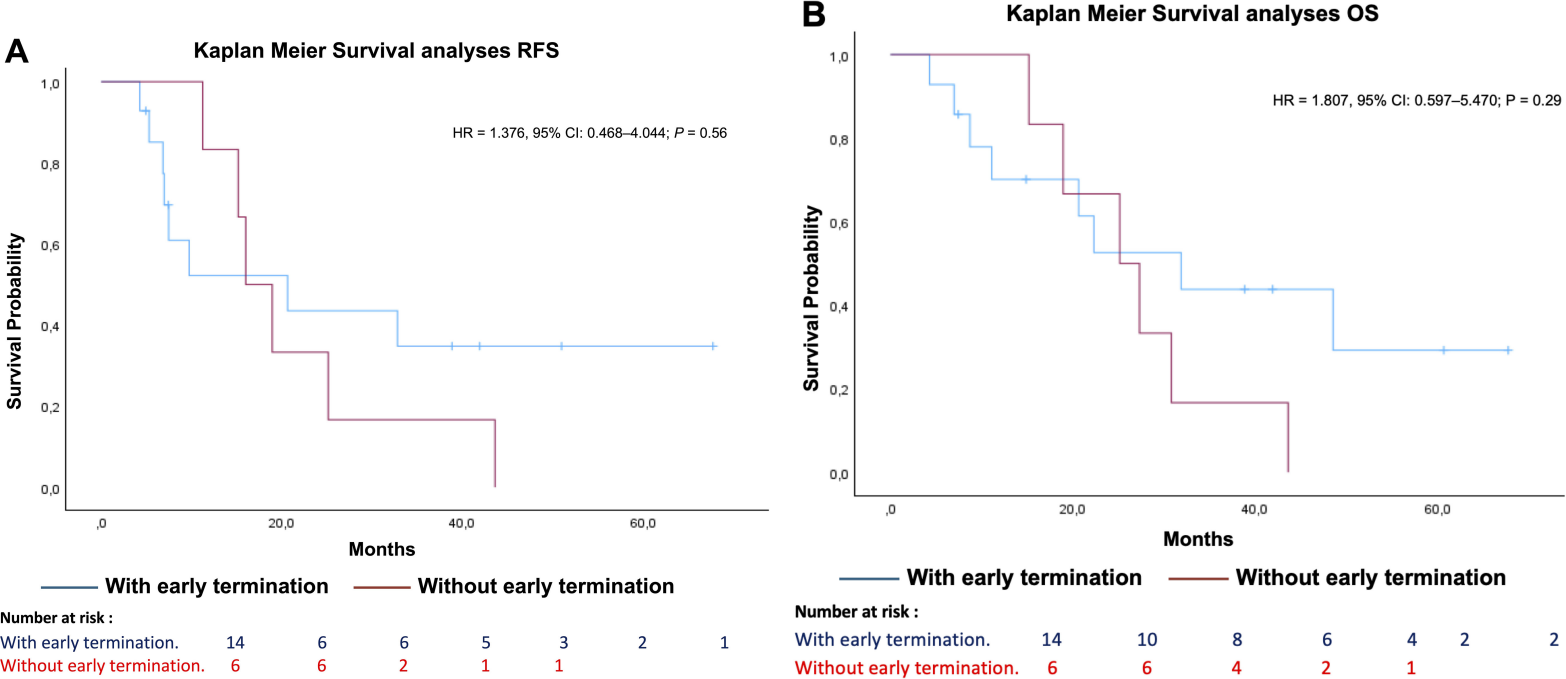
Recurrence free and overall survival with or without early termination. [**(A)** RFS; **(B)** OS; HR, Hazard ratio; CI, Confidence interval; RFS, Recurrence free survival; OS, Overall survival].

No significant differences in RFS were detected between patients who had a treatment break or dose reduction due to toxicity with early treatment termination (median RFS: 8.6 months) and those who had a break or reduction due to toxicity without early termination (median RFS: 17.4 months; HR = 1.376, 95% CI = 0.468–4.044; *P* = 0.56) (**Figure 5**). Similarly, there was no significant difference in overall survival (OS) between patients who experienced treatment-related toxicity leading to early termination (OS: 21.4 months) and those who completed treatment (median OS: 26.2 months; HR = 1.807, 95% CI = 0.597–5.470; *P* = 0.29).

## Discussion

BTC has a poor prognosis and is often diagnosed late, leaving most patients eligible only for palliative treatments. Among those eligible for resection, recurrence rates are high within 2 years after surgery. Despite the disappointing results of capecitabine as an adjuvant therapy and the absence of other effective treatments, this oral chemotherapy remains recommended for all patients regardless of CCA localization (7, 11).

In this retrospective observational study, we assessed the benefit of capecitabine in a real-world setting among consecutive patients who underwent BTC resection and were subsequently treated with capecitabine. Unfortunately, compared with surveillance, patients receiving capecitabine experienced similar recurrence rates and OS, even after adjustments and matching. We identified no benefits of capecitabine, even in the subgroup analyses.

To our knowledge, this is the first study to investigate the real-world benefit of capecitabine as an adjuvant treatment after BTC resection. The recently published study “Adjuvant Chemotherapy and Outcomes in Older Adult Patients With Biliary Tract Cancer” (12) examined the use of adjuvant chemotherapy in an elderly population but did not specifically evaluate the impact of capecitabine after curative-intent resection. Since the BILCAP clinical trial, no other study has specifically examined the real-world benefit of capecitabine in this setting. Real-world patients are not as selectively chosen as those in clinical trials and often present with worse general health. Moreover, real-world studies allow for faster treatment adaptations in cases of poor tolerance and can yield earlier results than those possible in clinical trials. Our center, an expert BTC treatment facility, manages patient care according to the individual benefit–risk ratio. The results of our study mirror those of the BILCAP trial, showing no significant real-world benefit of capecitabine. However, clinical trials remain the gold standard for generating clinical knowledge, and retrospective studies such as ours are inherently subject to biases.

Some data were absent in this study, highlighting a limitation of retrospective observational analyses. The missing information was related to patient compliance, treatment tolerance, and, in some cases, the cause of death. The coronavirus disease 2019 (COVID-19) pandemic adversely impacted patient care and follow-up. After curative treatment, some patients were monitored by local surgeons, and follow-up was conducted in peripheral centers once recovery from surgery was confirmed. This shift led to information loss regarding treatment tolerance and adherence. OS was measured from the date of surgery to the date of death, extracted either from patient records or the national death registry of the French National Institute for Statistical and Economic Studies. Because the cause of death is not included in this registry, this information was sometimes missing. Capecitabine, when used as an adjuvant treatment for BTC, provided a 5-year OS of 40% (13). Our study included a follow-up period of at least 1 year for patients receiving capecitabine; future updates will allow for analysis of longer-term outcomes. These issues inevitably affect the overall context of both prospective and retrospective studies.

Our study population exhibited biases typical of retrospective studies. Capecitabine was primarily prescribed to patients with advanced disease who were at higher risk of recurrence after surgery, consistent with previous guidelines. This could have led to a bimodal distribution of survival in the CC. Despite these worse prognostic factors, survival was similar in both groups. This may be because patients with a worse prognosis who received adjuvant chemotherapy had survival rates similar to those of patients who were not treated after surgery due to less advanced disease. Even after further analyses, capecitabine was considered ineffective as an adjuvant treatment. The Cox multivariable analysis, which adjusted for survival-associated variables, showed no significant differences in survival between the two cohorts. The PS matching analysis (1:1) enabled the comparison of cohorts with similar size and characteristics. The quality of the matching was assessed through the analysis of SMD before and after matching, confirming adequate balance between groups. Despite this adjustment, no benefit of capecitabine was observed in terms of OS or RFS.

As revealed in previous studies (14–17), patient adherence to treatment significantly affects outcomes across various clinical areas. Factors influencing adherence include the patient, as well as the healthcare provider and system (18). Even with good adherence, pharmacokinetics must be considered when evaluating the impact of a missed dose on treatment efficacy. Capecitabine has a short half-life of 45 min; thus, missing a dose leads to nearly complete elimination of the drug from the bloodstream, resulting in sub-therapeutic plasma concentrations. Poor adherence may reduce side effects but can also diminish therapeutic benefits, introducing bias into the study results. Although drug dosage monitoring is available at other centers, capecitabine blood level monitoring is not routinely performed or recommended in clinical practice. Blood level monitoring could be a powerful tool to improve patient management, offering a way to correlate therapeutic benefits, side effects, and capecitabine blood concentrations. Pharmacist follow-up could include capecitabine blood monitoring to adjust treatment for an optimal benefit–risk ratio and ensure patient compliance at home influencing treatment effectiveness and adverse event occurrence.

Nearly half of the patients in our study experienced significant side effects, mainly hand–foot syndrome. More than half of the patients had treatment interruptions, partly due to these side effects. Even for patients treated with the CP, no significant benefit was observed compared with those who received an AP. The proportion of patients who experienced significant side effects leading to treatment breaks or early discontinuation is concerning. In the BILCAP trial, 45% of patients did not complete the full eight cycles, and 46% required dose reductions. Our study showed similar results, with 53.5% of patients receiving an AP including treatment break or decrease in posology. These results highlight the practical challenges of maintaining treatment adherence and tolerance with capecitabine in real-world settings. Toxicity results previously cited, emphasize the need for structured multidisciplinary care pathways involving both patients and caregivers. In this context, clinical pharmacists play a pivotal role in optimizing the management of oral anticancer therapies. Since 2019, French regulations have mandated uracil blood testing before initiating capecitabine or fluorouracil, improving patient safety and reinforcing coordination between hospital and community pharmacies.

At Bordeaux University Hospital, dedicated multidisciplinary consultations have been progressively integrated into oncology care pathways since 2018, involving an oncologist, a nurse and a pharmacist. Pharmaceutical consultations provide patients with comprehensive written and verbal information on treatment management and supportive care, with a particular focus on nausea, diarrhea, and hand–foot syndrome—the most common dose-limiting toxicity observed in our cohort. Patients are educated to recognize early symptoms, and follow-up include remote adverse event and compliance management associated with community pharmacists coordination to adjust supportive measures and identify potential drug interactions. It is also an opportunity for these healthcare professionals to reemphasize the importance of patient adherence to treatment and its impact on treatment effectiveness. However, in our study, only 9 of the 43 patients receiving capecitabine benefited from a pharmaceutical consultation, underlining a gap between regulatory standards and real-life implementation.

The absence of systematic pharmacist involvement in the earlier years of our cohort may have influenced adherence, potentially offsetting part of the treatment benefit observed in controlled trials. Numerous prospective studies have shown that intensive pharmaceutical care improves adherence, reduces the incidence of severe adverse events, and enhances patient quality of life (9, 10, 14). Strengthening these structured pharmacist-led care pathways could therefore help maximize the therapeutic benefit of adjuvant capecitabine while minimizing toxicity-related interruptions.

In conclusion, our results align with the BILCAP findings, showing no clear benefit or efficacy of capecitabine as an adjuvant therapy after BTC resection. Although the BILCAP trial suggested a benefit upon reanalysis, this was not observe in this work. A small benefit cannot be excluded, as real-world populations are heterogeneous and patient adherence and toxicity management are less standardized, which can affect treatment effectiveness. Detecting such a benefice is further limited by the smaller size of our cohort compared to the BILCAP trial. A larger, multicentric study could increase the statistical power and help confirm these preliminary findings. Since this trial, several studies have examined the benefit of adjuvant treatment after BTC surgery (4). The STAMP phase II clinical trial compared the combination of gemcitabine and cisplatin with capecitabine alone in patients with eCCA who had node-positive disease and underwent curative surgery involving R0 or R1 resection. The results showed no additional benefit of this combination over capecitabine (19). The SWOG phase II intergroup trial investigated the benefit of gemcitabine plus capecitabine followed by capecitabine and radiotherapy in patients with eCCA and GBC. The results suggested a benefit, but the absence of a control arm limited the interpretation (20). A meta-analysis of 20 studies supported the use of adjuvant therapy in patients with BTC exhibiting high-risk features (4). However, the lack of randomized data highlights the need for prospective multicenter studies, especially focusing on patients with a high risk of recurrence. Future trials could evaluate new targeted therapies or innovative combinations with better tolerability, which are currently used in advanced stages of disease. New care pathways, including molecular screening at diagnosis and pharmaceutical consultations at treatment initiation, also could be developed to optimize patient outcomes.

## Data Availability

The raw data supporting the conclusions of this article will be made available by the authors, without undue reservation.
